# Comparative analysis of adductor canal block combined with iPACK block versus femoral combined with sciatic nerve blocks: a propensity score matched study

**DOI:** 10.1186/s12871-025-03112-z

**Published:** 2025-05-16

**Authors:** Chao-Hsien Sung, Jen-Hao Liu, Chi-Feng Hung, Chun-Hsien Fu

**Affiliations:** 1 Division of Anesthesiology, Fu Jen Catholic University Hospital, Fu Jen Catholic University, No.69, Guizi Rd., Taishan Dist, New Taipei City, 24352 Taiwan; 2 Department of Anesthesiology, National Taiwan University Hospital, Taipei City, Taiwan; 3 School of Medicine, Fu Jen Catholic University, New Taipei City, Taiwan; 4 PhD Program in Pharmaceutical Biotechnology, Fu Jen Catholic University, New Taipei City, Taiwan

**Keywords:** Adductor canal block, iPACK, Total knee arthroplasties, Postoperative analgesia, Motor function recoveries, Nerve block

## Abstract

**Background:**

Nerve blocks are effective in reducing postoperative opioid use and enhancing rehabilitation following total knee arthroplasty. However, few studies compare the analgesic efficacy and functional recovery of adductor canal block (ACB) combined with infiltration between the popliteal artery and the capsule of the knee (iPACK) versus sciatic and femoral nerve blocks (S + F). This study hypothesized that ACB combined with iPACK (A + I) provides comparable analgesia to S + F with superior motor recovery.

**Methods:**

Data were obtained from a prospectively maintained acute pain service database. After exclusion criteria were applied, 126 patients were analyzed. Propensity-score matching balanced baseline characteristics between the A + I and S + F groups. Numeric rating scale (NRS) scores at different time points were primary outcome. Motor function analysis, including the motor blockade, maximum flexion angle and time to ambulation were secondary outcomes.

**Results:**

After propensity score matching, patients in the A + I group reported significantly lower NRS pain scores in the post-anesthetic care unit (1.00 ± 0.72 vs. 1.52 ± 1.34; *P* = 0.026) and on postoperative day 1 at rest (0.66 ± 0.71 vs. 1.07 ± 0.95; *P* = 0.025) and during movement (1.75 ± 0.75 vs. 2.43 ± 1.19; *P* = 0.002). Movement-associated pain on postoperative day 2 was also lower in the A + I group (1.45 ± 0.66 vs. 2.34 ± 0.91; *P* < 0.001). The A + I group exhibited significantly less motor blockade (*P* < 0.001) and achieved earlier ambulation (1551.75 ± 379.98 vs. 2031.95 ± 764.77 min; *P* < 0.001).

**Conclusions:**

The A + I regimen demonstrated superior analgesic efficacy, reduced motor blockade, and earlier ambulation compared to S + F in TKA patients. These findings support the use of A + I for improved recovery.

**Trial registration:**

This trial was registered before collection of data and analysis at ClinicalTrials.gov (NCT06521619). Date of registration: 2024-07-26.

## Introduction

Total knee arthroplasty (TKA) is a widely performed surgical procedure wherein the knee joint is replaced with a prosthetic implant to mitigate pain and functional impairment resulting from conditions such as osteoarthritis, rheumatoid arthritis, and posttraumatic arthritis [1, 2]. TKA enhances the quality of life of patients, particularly older patients. It has major implications for pain management, mobility, and overall well-being. Approximately 40% of all individuals aged above 65 years develop knee osteoarthritis, contributing to the rising prevalence of TKA [1].

Post-TKA rehabilitation focuses on restoring knee function and mobility. After surgery, patients are encouraged to start moving their knees to prevent joint stiffness and complications such as blood clotting. Physiotherapists assist with gentle exercises aimed at improving range of motion (ROM) and strength. Postoperative pain often undermines rehabilitation programs, delays functional recovery, and increases thromboembolism risks [3].

Multimodal analgesia is achieved after surgery through combination of systemic medications (e.g., opioids, nonsteroidal anti-inflammatory drugs, acetaminophen, and gabapentin), neuraxial analgesia, peripheral nerve block, or articular local anesthetic infiltration. Nerve block is an essential component of multimodal analgesia. Perioperative nerve block can reduce intraoperative and postoperative opioid use, alleviate pain, improve adherence to rehabilitation programs, shorten hospital stay, and enhance patient satisfaction [4–6].

Nerve block regimens proposed for the perioperative management of patients undergoing TKA include femoral nerve block, sciatic nerve block, adductor canal block (ACB), infiltration between the popliteal artery and the capsule of the knee (iPACK), and periarticular local infiltration [7]. Combining femoral nerve block with sciatic nerve block results in adequate surgical anesthesia and enhances postoperative analgesia [8]. However, femoral nerve block can lead to quadriceps weakness, which delays rehabilitation and increases fall risks [9, 10]. ACB has gained popularity for postoperative analgesia because of its opioid- and motor-sparing effects [11–13]. This nerve block regimen exerts analgesic effects similar to those of femoral nerve block [14]. Previous literature suggests that iPACK primarily targets the small branches of the genicular nerves and the popliteal plexus, which innervate the posterior aspect of the knee [15].

Few studies have compared the efficacy and functional recovery effects of ACB combined with iPACK (A + I) and sciatic combined with femoral nerve blocks (S + F). In this study, we hypothesized that, compared with the single-shot S + F regimen, the single-shot A + I regimen would lead to similar pain control, lower motor blockade, and faster functional recovery after TKA. This retrospective study was conducted to test this hypothesis.

## Methods

### Study cohort

This study was approved by the Institutional Review Board of Fu Jen Catholic University Hospital, Taiwan (approval number: FJUH113365, approval date: June 26, 2024). Given the retrospective nature of this study, the requirement for informed consent was waived by the board. Patient information such as name, chart number, and surgery date were omitted during data collection to ensure privacy. Data were collected from a prospectively maintained acute pain services database, which includes information on all patients who received nerve blocks for postoperative analgesia in our department. Patients who underwent TKA and received nerve block for postoperative analgesia between January 1, 2022, and December 31, 2023, were included for analysis. The exclusion criteria were as follows: not undergoing general anesthesia for surgery and not receiving the S + F or A + I regimen. A patient-controlled analgesia (PCA) pump was prescribed for a duration of 2 days postoperatively in patient who received nerve block. Postoperative data on pain during movement, pain at rest, degree of motor blockade, and opioid consumption of PCA were routinely collected by nurse anesthetists daily. These data were reviewed daily by a dedicated team to identify any missing or incorrect information. Data on the maximum passive ROM angle at postoperative day 1 and earliest ambulation time were extracted from the patients’ medical records. The study adheres to applicable STROBE guidelines.

### Anesthesia induction and emergence

Anesthesia was induced by attending anesthesiologists and nurse anesthetists. The medications used for anesthesia induction and nerve block were determined by the attending anesthesiologists, who also determined the nerve block regimen. Commonly used agents such as propofol (2 mg/kg), thiamylal (3–5 mg/kg), cisatracurium (0.15 mg/kg), rocuronium (0.5–1 mg/kg), fentanyl (1–2 µg/kg), glycopyrrolate (0.2 mg), dexamethasone (4 mg), or lidocaine (1 mg/kg) were used to induce anesthesia. After surgery, muscle relaxation was reversed using neostigmine (0.04–0.07 mg/kg) and glycopyrrolate or sugammadex (2–4 mg/kg) and patients were extubated after train-of-four ratio greater than 0.9. Patients were transferred to postoperative care unit (PACU) for postoperative observation.

### Sciatic and femoral nerve blocks

Femoral nerve block was performed with the patient in the supine position. Under sterile conditions, a linear transducer was placed along the line of the inguinal crease, the femoral nerve lateral to the common femoral artery was identified. Subsequently, a 20-G needle was inserted laterally to medially by using an in-plane technique. To avoid accidental vascular injection, aspiration was performed before locally injecting 10–15 mL of the anesthetic agent.

Sciatic nerve block was similarly performed with the patient in the supine position, with the target leg elevated. Under sterile conditions, a linear transducer was placed on the popliteal fossa, the popliteal sciatic nerve was identified as a hyperechoic round structure at the posterior aspect of the thigh. Subsequently, a 20-G needle was inserted from the lateral thigh by using an in-plane technique. After careful aspiration, 20 mL of the anesthetic agent was injected adjacent to the sciatic nerve.

### Adductor canal block and iPACK

ACB was performed with the patient in the supine position, with the legs in external rotation and the knees in slight flexion. Under sterile conditions, a linear transducer was placed transversely at the junction between the middle and distal third of the thigh, the saphenous nerve in the adductor canal was identified. Subsequently, a 20-G needle was inserted laterally to medially by using an in-plane technique. To avoid accidental vascular injection, aspiration was performed before locally injecting 10–15 mL of the anesthetic agent.

iPACK was performed with the hips in external rotation and knees in slight flexion. Under sterile condition, a curved transducer was placed at the popliteal fossa, the popliteal arteries in the short axis were identified. The probe was gradually moved distally to locate the space between the popliteal arteries and posterior aspects of femur shaft. A 20-G needle was advanced into this region. After careful aspiration, 20 mL of the anesthetic agent was locally injected under sterile conditions.

### PCA regimen

The PCA regimen and settings were determined by the attending anesthesiologists. The most commonly prescribed PCA regimen was fentanyl at a concentration of 10 µg/mL.

### Study outcomes

The primary study outcome was postoperative pain, which was evaluated using the numeric rating scale (NRS) at PACU, on postoperative day 1 (POD1) and on postoperative day 2 (POD2). Pain was assessed during movement and at rest. Motor blockade was evaluated by the modified Bromage motor blockade score (0–3). The maximum flexion angle was recorded on POD1. The earliest ambulation time was defined as the interval between the end of surgery and the first instance of ambulation after surgery. Satisfaction score represents the overall level of satisfaction with postoperative pain relief, where 1 indicates the lowest level of satisfaction and 5 indicates the highest. NRS evaluation, PCA dose consumption, satisfaction score and motor blockade were extracted from records of routine postoperative visits by acute pain services team in our department. Maximum flexion angle and earliest ambulation time were extracted from medical records.

### Statistical analysis

Dichotomous data were analyzed using the chi-square test. Normally distributed data were analyzed using Student’s *t* test. Nonparametric ordinal data were analyzed using the Mann–Whitney *U* test. *P* values less than 0.05 indicated statistical significance. To address selection bias, a propensity score matching (PSM) analysis was performed to estimate the conditional probability of receiving a specific treatment based on a set of observed covariates. This analysis employed a logistic regression model, where the type of nerve block (A + I vs. S + F) was regressed on key variables such as age, sex, body mass index (BMI), and the local anesthetic regimen, all considered critical to the evaluation of outcomes. Using this model, a propensity score was calculated for each patient. Patients in the A + I group were matched with those in the S + F group with similar propensity scores in a 1:1 manner using nearest neighbor matching method. All statistical analyses were conducted using IBM SPSS Statistics version 26.0 (IBM, Armonk, NY, USA).

## Results

### Cohort characteristics

**Fig. 1:**
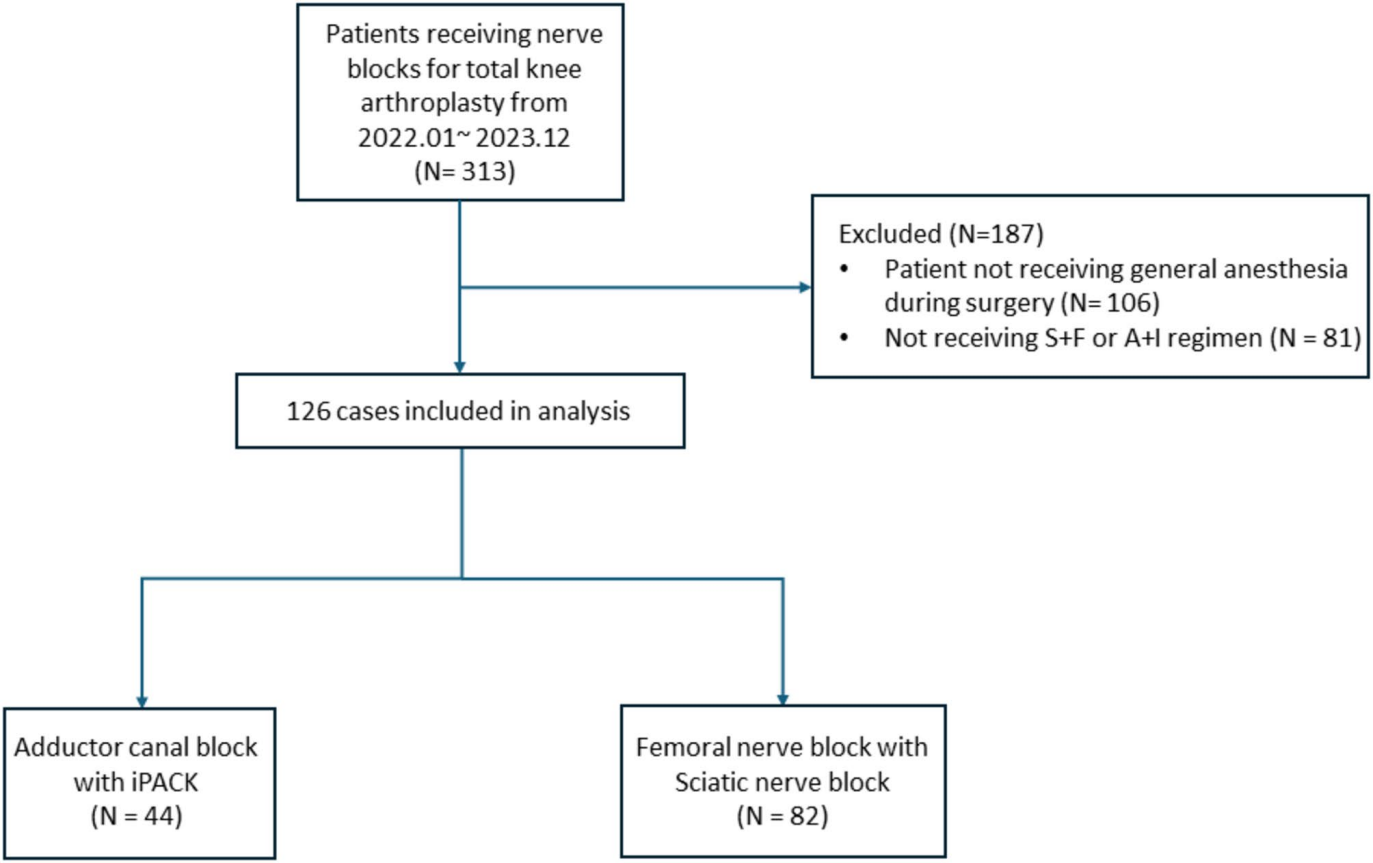
Diagram for patient inclusion and exclusion

Between January 1, 2022, and December 31, 2023, a total of 314 patients in our hospital received nerve block for post-TKA analgesia. After the exclusion of ineligible patients, 126 patients were included in the final analysis (Fig. 1). A total 44 patients received the A + I regimen (A + I group), whereas 82 patients received the S + F regimen (S + F group). No significant between-group difference was observed in baseline demographic characteristics (Table 1).

**Table 1:** Demographic characteristics of the study groups before propensity score matching. Values are mean (SD) or number (proportion)

	ACB + iPACK *N* = 44	Sciatic + Femoral *N* = 82	*P*
Age (y)	69.43 (9.38)	69.77 (8.10)	0.834
Male Sex (N)	12 (27.27%)	16 (19.51%)	0.318
BMI^*^ (kg/m^2^)	28.65 (3.95)	28.29 (5.23)	0.696
Duration of Anesthesia (min)	179.36 (17.59)	184.51 (23.32)	0.167
Regimen of block			
0.5% Ropivacaine	44 (100%)	69 (84.14%)	
0.25% Bupivacaine	0 (0%)	2 (2.43%)	
2% lidocaine with 1% ropivacaine 1% lidocaine + 0.5% ropivacaine	0 (0%) 0 (0%)	5 (6.10%) 6 (7.33%)	0.051
Total amount of local anesthetics (mL)	34.86 (5.55)	39.77 (5.80)	**< 0.001**
Acetaminophen given (N)	44 (100%)	80 (97.56%)	0.296
Skin rash (N)	0 (0%)	0 (0%)	
Postoperative nausea and vomiting (N)	0 (0%)	2 (2.44%)	0.296
Urinary retention (N)	0 (0%)	0 (0%)	
Satisfaction score (N)			
1	0 (0%)	0 (0%)	
2	0 (0%)	0 (0%)	
3	0 (0%)	0 (0%)	
4	2 (4.55%)	2 (2.44%)	
5	42 (95.45%)	80 (97.56%)	0.520
Length of hospital stay (Days)	6.32 (1.03)	6.79 (1.38)	**0.047**

^*^BMI: Body Mass Index

### Between-group differences before PSM

Before PSM, borderline differences (*P* = 0.051) in the nerve block regimen were observed between the two groups. All patients in the A + I group received 0.5% ropivacaine, whereas only 69 out of 82 patients in the S + F group received 0.5% ropivacaine. Among the remaining patients, 2 received 0.25% bupivacaine and 11 received other nerve block agents.

Significant between-group differences were observed in the level of pain experienced by patients in the PACU (Table 2). The average NRS score was significantly lower in the A + I group than in the S + F group (1.00 ± 0.75 vs. 1.46 ± 1.41; *P* = 0.016). In addition, significant between-group differences in pain were observed at rest and during movement on POD1. The average NRS score for pain at rest was significantly lower in the A + I group than in the S + F group (0.66 ± 0.71 vs. 1.10 ± 1.23; *P* = 0.032). Similarly, the average NRS score for pain during movement was significantly lower in the A + I group than in the S + F group (1.75 ± 0.75 vs. 2.40 ± 1.40; *P* = 0.001). On POD2, the average NRS score for pain at rest was lower, though nonsignificantly, in the A + I group than in the S + F group (0.45 ± 0.55 vs. 0.61 ± 0.66; *P* = 0.186). The average NRS score for pain during movement at POD2 was significantly lower in the A + I group than in the S + F group (1.45 ± 0.66 vs. 2.21 ± 0.99; *P* < 0.001).

**Table 2:** Pain scores of the study groups before propensity score matching. Values are mean (SD) or number (proportion)

	ACB + iPACK *N* = 44	Sciatic + Femoral *N* = 82	*P*
NRS^*^ at PACU ^†^	1.00 (0.715)	1.46 (1.41)	**0.016**
NRS at POD ^‡^ 1 while moving	1.75 (0.75)	2.40 (1.40)	**0.001**
NRS at POD1 while resting	0.66 (0.71)	1.10 (1.23)	**0.032**
NRS at POD2 while moving	1.45 (0.66)	2.21 (0.99)	**< 0.001**
NRS at POD2 while resting	0.45 (0.55)	0.61 (0.66)	0.186
Intraoperative fentanyl (µg)	136.02 (46.41)	97.44 (32.87)	**< 0.001**
Intraoperative ketorolac, No.	20 (45.45%)	2 (2.44%)	**< 0.001**
Fentanyl consumption at POD1 (µg)	99.50 (79.83)	173.38 (152.22)	**0.001**
Fentanyl consumption at POD2 (µg)	112.27 (79.08)	186.15 (137.41)	**< 0.001**
Readjustment of PCA^§^ setting for pain rescue, No.	0 (0%)	4 (4.88%)	0.137

^*^ NRS, Numeric rating scale; ^†^PACU, postanesthesia care unit; ^‡^POD, postoperative day; ^§^PCA, patient-controlled analgesia

The amount of fentanyl administered intraoperatively was significantly higher in the A + I group than in the S + F group (136.02 ± 46.41 vs. 97.44 ± 32.87 µg; *P* < 0.001). On POD1, the amount of fentanyl administered was significantly lower in the A + I group than in the S + F group (99.50 ± 79.83 vs. 173.38 ± 152.22 µg; *P* = 0.001). On POD2, the amount of fentanyl administered was significantly lower in the A + I group than in the S + F group (112.27 ± 79.08 vs. 186.15 ± 137.41 µg; *P* < 0.001). Readjustment of the PCA regimen for pain control was not required for the A + I group, but it was necessary for four patients in the S + F group (between-group difference, *P* = 0.137). The number of patients who received intraoperative ketorolac was significantly higher in the A + I group than in the S + F group (20 [45.45%] vs. 2 [2.44%]; *P* < 0.001).

Before PSM, analysis of motor function revealed significant differences in the extent of motor blockade between the two groups (Table 3). The number of patients with no postoperative motor blockade was significantly higher in the A + I group than in the S + F group (42 [95.45%] vs. 26 [31.71%]; *P* < 0.001). On POD1, a significant difference in ROM was observed between the two groups. The average maximum flexion angle was significantly higher in the A + I group than in the S + F group (75.57° ± 11.22° vs. 67.20° ± 15.24°; *P* = 0.002). In addition, the earliest ambulation time was significantly shorter in the A + I group than in the S + F group (1551.75 ± 379.98 vs. 2114.96 ± 770.52 min; *P* < 0.001).

**Table 3:** Motor function recovery in the study groups before propensity score matching values are mean (SD) or number (proportion)

	ACB + iPACK *N* = 44	Sciatic + Femoral *N* = 82	*P*
Modified Bromage Scale, Grade			
0	42 (95.45%)	26 (31.71%)	
1	1 (2.27%)	42 (51.22%)	
2	1 (2.27%)	13 (15.85%)	
3	0 (0%)	1 (1.22%)	**< 0.001**
Range of motion at POD^*^ 1 (degrees)	75.57 (11.22)	67.20 (15.24)	**0.002**
Earliest ambulation time (min) ^†^	1551.75 (379.98)	2114.96 (770.52)	**< 0.001**

^**#**^ POD, postoperative day

^†^ Defined as the interval between surgery and the first instance of postoperative ambulation

A minor between-group difference was observed in the length of hospital stay. The average length of stay was significantly shorter in the A + I group than in the S + F group (6.32 ± 1.03 vs. 6.79 ± 1.38 days; *P* = 0.047).

### Between-group differences after PSM

To eliminate potential bias, PSM was performed with age, sex, and nerve block regimen as variables. After PSM, no significant between-group differences were observed in baseline demographic characteristics. All patients received 0.5% ropivacaine for nerve block (Table 4).

**Table 4:** Demographic characteristics of the study groups after propensity score matching. Values are mean (SD) or number (proportion)

	ACB + iPACK *N* = 44	Sciatic + Femoral *N* = 44	*P*
Age (y)	69.43 (9.38)	70.86 (7.48)	0.431
Male Sex (N)	12 (27.27%)	12 (27.27%)	1.000
BMI^*^ (kg/m^2^)	28.65 (3.95)	28.93 (4.56)	0.757
Duration of Anesthesia (min)	179.36 (17.59)	186.57 (25.64)	0.128
Regimen of block (N)			
0.5% Ropivacaine	44 (100%)	44 (100%)	
0.25% Bupivacaine	0 (0%)	0 (0%)	
Other Regimen	0 (0%)	0 (0%)	
Total amount of local anesthetics (mL)	34.86 (5.55)	38.75 (4.96)	**0.001**
Acetaminophen given (N)	44 (100%)	44 (100%)	
Skin rash (N)	0 (0%)	0 (0%)	
Postoperative nausea and vomiting (N)	0 (0%)	1 (2.27%)	0.315
Urinary retention (N)	0 (0%)	0 (0%)	
Satisfaction score (N)			
1	0 (0%)	0 (0%)	
2	0 (0%)	0 (0%)	
3	0 (0%)	0 (0%)	
4	2 (4.55%)	0 (0%)	
5	42 (95.45%)	44 (100%)	0.153
Length of hospital stay (Days)	6.32 (1.03)	6.68 (1.29)	0.147

^*^BMI: Body Mass Index

Significant between-group differences were observed in the level of pain experienced by the patients in the PACU (Table 5). The average NRS score was significantly lower in the A + I group than in the S + F group (1.00 ± 0.72 vs. 1.52 ± 1.34; *P* = 0.026). In addition, significant between-group differences were observed in pain at rest and during movement on POD1. The average NRS score for pain at rest was significantly lower in the A + I group than in the S + F group (0.66 ± 0.71 vs. 1.07 ± 0.95; *P* = 0.025). The average NRS score for pain during movement was significantly lower in the A + I group than in the S + F group (1.75 ± 0.75 vs. 2.43 ± 1.19; *P* = 0.002). On POD2, the average NRS score for pain at rest was similar between the A + I and S + F groups (0.45 ± 0.55 vs. 0.52 ± 0.63; *P* = 0.589). The average NRS score for pain during movement on POD2 was significantly lower in A + I group than in the S + F group (1.45 ± 0.66 vs. 2.34 ± 0.91; *P* < 0.001).

**Table 5:** Pain scores of the study groups after propensity score matching values are mean (SD) or number (proportion)

	ACB + iPACK *N* = 44	Sciatic + Femoral *N* = 44	*P*
NRS^*^ at PACU ^†^	1.00 (0.72)	1.52 (1.34)	**0.026**
NRS at POD ^‡^ 1 while moving	1.75 (0.75)	2.43 (1.19)	**0.002**
NRS at POD1 while resting	0.66 (0.71)	1.07 (0.95)	**0.025**
NRS at POD2 while moving	1.45 (0.66)	2.34 (0.91)	**< 0.001**
NRS at POD2 while resting	0.45 (0.55)	0.52 (0.63)	0.589
Intraoperative fentanyl (µg)	136.02 (46.41)	101.59 (34.79)	**< 0.001**
Intraoperative ketorolac, No.	20 (45.45%)	1 (2.27%)	**< 0.001**
Fentanyl consumption at POD1 (µg)	99.50 (79.83)	144.97 (122.06)	**0.042**
Fentanyl consumption at POD2 (µg)	112.27 (79.08)	179.82 (128.86)	**0.004**
Readjustment of PCA^§^ setting for pain rescue, No.	0 (0%)	1 (2.27%)	0.315

^*^ NRS, Numeric rating scale; ^†^PACU, postanesthesia care unit; ^‡^POD, postoperative day; ^§^PCA, patient-controlled analgesia

The amount of fentanyl administered intraoperatively was significantly higher in the A + I group than in the S + F group (136.02 ± 46.41 vs. 101.59 ± 34.79 µg; *P* < 0.001). On POD1, the amount of fentanyl administered was significantly lower in the A + I group than in the S + F group (99.50 ± 79.83 vs. 144.97 ± 122.06 µg; *P* = 0.042). Similarly, on POD2, the amount of fentanyl administered was significantly lower in the A + I group than in the S + F group (112.27 ± 79.08 vs. 179.82 ± 128.86 µg; *P* = 0.004). Readjustment of the PCA regimen for pain control was not required in the A + I group, but it was necessary for one patient in the S + F group (between-group difference, *P* = 0.315). The number of patients who received intraoperative ketorolac was significantly higher in the A + I group than in the S + F group (20 [45.45%] vs. 1 [2.27%]; *P* < 0.001).

After PSM, analysis of motor function revealed significant differences in the extent of motor blockade between the two groups (Table 6). The number of patients with no postoperative motor blockade was significantly higher in the A + I group than in the S + F group (42 [95.45%] vs. 8 [18.18%]; *P* < 0.001). On POD1, a significant difference was observed in ROM between the two groups. The average maximum flexion angle was significantly higher in the A + I group than in the S + F group (75.57° ± 11.22° vs. 69.89° ± 15.19°; *P* = 0.049). In addition, the earliest ambulation time was significantly shorter in the A + I group than in the S + F group (1551.75 ± 379.98 vs. 2031.95 ± 764.77 min; *P* < 0.001).

**Table 6:** Motor function recovery in the study groups after propensity score matching values are mean (SD) or number (proportion)

	ACB + iPACK *N* = 44	Sciatic + Femoral *N* = 44	*P*
Modified Bromage Scale, Grade			
0	42 (95.45%)	8 (18.18%)	
1	1 (2.27%)	26 (59.09%)	
2	1 (2.27%)	10 (22.72%)	
3	0 (0%)	0 (0%)	**< 0.001**
Range of motion at POD^*^ 1 (degrees)	75.57 (11.22)	69.89 (15.19)	**0.049**
Earliest ambulation time (min) ^†^	1551.75 (379.98)	2031.95 (764.77)	**< 0.001**

^**#**^ POD, postoperative day

^†^ Defined as the interval between surgery and the first instance of postoperative ambulation

No significant between-group difference was observed in the length of hospital stay (*P* = 0.147).

### Subgroup analysis

To further exclude the influence of intraoperative analgesic use, we performed subgroup analysis excluding intraoperative fentanyl usage more than 150 µg and ketorolac usage. We observed no significant difference in baseline characteristics between groups (Table 7).

**Table 7:** Demographic characteristics of subgroup analysis excluding Fentanyl usage more than 150 µg and Ketorolac. Values are mean (SD) or number (proportion)

	ACB + iPACK *N* = 22	Sciatic + Femoral *N* = 80	*P*
Age (y)	69.86 (7.754)	69.99 (8.062)	0.949
Male Sex (N)	8 (36.4%)	16 (20.0%)	0.109
BMI^*^ (kg/m^2^)	28.48 (3.79)	28.36 (5.27)	0.921
Duration of Anesthesia (min)	184.45 (16.83)	184.54 (23.37)	0.988
Regimen of block			
0.5% Ropivacaine	22 (100%)	67 (83.75%)	
0.25% Bupivacaine	0 (0%)	2 (2.50%)	
2% lidocaine with 1% ropivacaine	0 (0%)	5 (6.25%)	0.251
1% lidocaine + 0.5% ropivacaine	0 (0%)	6 (7.50%)	
Total amount of local anesthetics (mL)	34.86 (7.05)	40.01 (5.65)	**0.004**
Acetaminophen given (N)	22 (100%)	78 (97.50%)	0.454
Skin rash (N)	0 (0%)	0 (0%)	
Postoperative nausea and vomiting (N)	0 (0%)	2 (2.50%)	0.454
Urinary retention (N)	0 (0%)	0 (0%)	
Satisfaction score (N)			
1	0 (0%)	0 (0%)	
2	0 (0%)	0 (0%)	
3	0 (0%)	0 (0%)	
4	1 (4.55%)	1 (1.25%)	
5	21 (95.45%)	79 (98.75%)	0.323
Length of hospital stay (Days)	6.36 (0.95)	6.86 (1.32)	0.101

^*^BMI: Body Mass Index

There is a marginal difference observed in the NRS score at PACU between A + I group and S + F group (1.09 ± 0.75 vs. 1.49 ± 1.41; *P* = 0.082). On POD1, the average NRS score during movement is significantly lower in A + I group than S + F group (1.82 ± 0.85 vs. 2.44 ± 1.40; *P* = 0.012). As for the average NRS score at rest on POD1, A + I group demonstrated a lower NRS score, though non-significant, than S + F group (0.59 ± 0.73 vs. 1.13 ± 1.24; *P* = 0.056). On POD2, the average NRS score in A + I group during movement is significantly lower than in S + F group (1.27 ± 0.63 vs. 2.23 ± 0.99; *P* < 0.001). However, the average NRS score at rest on POD2 showed marginal difference between A + I group and S + F group (0.41 ± 0.50 vs. 0.63 ± 0.66; *P* = 0.160). We observed no statistically significant difference in intraoperative fentanyl usage between groups (*P* = 0.401) (Table 8).

**Table 8:** Pain scores of subgroup analysis excluding Fentanyl usage more than 150 µg and Ketorolac. Values are mean (SD) or number (proportion)

	ACB + iPACK *N* = 22	Sciatic + Femoral *N* = 80	*P*
NRS^*^ at PACU ^†^	1.09 (0.75)	1.49 (1.41)	0.082
NRS at POD ^‡^ 1 while moving	1.82 (0.85)	2.44 (1.40)	**0.012**
NRS at POD1 while resting	0.59 (0.73)	1.13 (1.24)	0.056
NRS at POD2 while moving	1.27 (0.63)	2.23 (0.99)	**< 0.001**
NRS at POD2 while resting	0.41 (0.50)	0.63 (0.66)	0.160
Intraoperative fentanyl (µg)	105.00 (33.49)	98.31 (32.74)	0.401
Fentanyl consumption at POD1 (µg)	93.73 (88.31)	176.09 (153.04)	**0.018**
Fentanyl consumption at POD2 (µg)	114.91 (71.00)	188.05 (138.45)	**0.001**
Readjustment of PCA^§^ setting for pain rescue, No.	0 (0%)	4 (5.0%)	0.285

^*^ NRS, Numeric rating scale; ^†^PACU, postanesthesia care unit; ^‡^POD, postoperative day; ^§^PCA, patient-controlled analgesia

There is a significant difference observed in fentanyl consumption on POD1 between A + I group and S + F group (93.73 ± 88.31 vs. 176.09 ± 153.04 µg; *P* = 0.018). Furthermore, on POD2, fentanyl consumption is significantly lower in A + I group compared to S + F group (114.91 ± 71.00 vs. 188.05 ± 138.45 µg; *P* = 0.001). Readjustment of the PCA regimen for pain control was not required for the A + I group, but it was necessary for four patients in the S + F group (between-group difference, *P* = 0.285).

Analysis of modified bromage scale revealed significant difference between two groups (*P* < 0.001) (Table 9). On POD1, a statistically significant difference was observed in ROM between two groups. The average maximum flexion angle is significantly higher in the A + I group than in the S + F group (75.45° ± 11.33° vs. 68.25° ± 13.27°; *P* = 0.022). In addition, the earliest ambulation time was significantly shorter in the A + I group than in the S + F group (1342.23 ± 210.18 vs. 1944.30 ± 773.50 min; *P* < 0.001).

**Table 9:** Motor function recovery in subgroup analysis excluding Fentanyl usage more than 150 µg and Ketorolac. Values are mean (SD) or number (proportion)

	ACB + iPACK *N* = 22	Sciatic + Femoral *N* = 80	*P*
Modified Bromage Scale, Grade			
0	21 (95.45%)	24 (30.00%)	
1	1 (4.55%)	42 (52.50%)	
2	0 (0%)	13 (16.25%)	
3	0 (0%)	1 (1.25%)	**< 0.001**
Range of motion at POD^*^ 1 (degrees)	75.45 (11.33)	68.25 (13.27)	**0.022**
Earliest ambulation time (min) ^†^	1342.23 (210.18)	1944.30 (773.50)	**< 0.001**

^**#**^ POD, postoperative day

^†^ Defined as the interval between surgery and the first instance of postoperative ambulation

No significant between-group difference was observed in the length of hospital stay (*P* = 0.101).

## Discussion

In this study, we compared the analgesic efficacy of the A + I and S + F nerve block regimens. Our findings indicated that the A + I regimen is an effective analgesic approach whose efficacy surpasses that of the S + F regimen. Combination of sciatic nerve block and femoral nerve block provide adequate surgical anesthesia [16]. In patients undergoing TKA, combining the sciatic and femoral nerve blocks is more effective than femoral nerve block alone or periarticular local infiltration [17, 18]. However, the motor blockade resulting from this combined regimen impedes early rehabilitation and ambulation [13].

iPACK nerve block, a novel ultrasound-guided technique, involves distributing local anesthetics to anesthetize the knee’s posterior aspect by targeting the small articular sensory branches of the popliteal plexus and obturator nerve [19, 20]. This approach provides an analgesic effect without causing motor blockade [5, 15, 21, 22]. ACB primarily targets the saphenous nerve and is regarded as a motor-sparing technique leading to less opioid consumption and less opioid related adverse events [23–26]. Few studies have compared the A + I and S + F regimens in terms of efficacy. In our study, the patients’ average NRS score in the PACU was significantly lower in the A + I group than in the S + F group (Before PSM, *P* = 0.016; after PSM, *P* = 0.026). This difference may be attributable to the fact that the amount of fentanyl administered intraoperatively and the number of patients receiving intraoperative ketorolac were significantly higher in the A + I group than in the S + F group (fentanyl amount: 136.02 ± 46.41 vs. 97.44 ± 32.87 µg, *P* < 0.001; patients receiving ketorolac: 20 [45.45%] vs. 2 [2.44%], *P* < 0.001). The differences in the total intraoperative doses of fentanyl and ketorolac may explain the lower NRS scores observed in the A + I group compared to S + F group in PACU. Subgroup analysis excluding intraoperative fentanyl usage more than 150 µg revealed no significant difference on average PACU NRS score further confirmed the difference we observed may be attributed to the influence of higher intraoperative fentanyl usage.

On POD1, the average NRS scores for pain during movement and pain at rest were significantly lower in the A + I group than in the S + F group. The A + I group also exhibited reduced fentanyl use. These findings suggest that the A + I regimen is more effective than the S + F regimen in achieving postoperative analgesia. This analgesic effect extended to POD2, wherein the average NRS score for pain during movement was significantly lower in the A + I group than in the S + F group. However, the average NRS score for pain at rest did not significantly differ between the two groups. Li et al. argued that pain after lower-extremity surgery, particularly knee surgery, peaks on POD1 [3]. Pain intensity typically decreases by POD5 [27], as pain due to swelling, surgical trauma, and inflammation gradually subsides. Inadequate pain control delays functional recovery and increases complication risks [19, 28]. The subgroup analysis revealed that the NRS score on both POD1 and POD2 while moving was significantly lower in the A + I group. In addition, there are marginal differences observed in the average NRS score at rest on PACU (*P* = 0.082), POD1 (*P* = 0.056) and POD2 (*P =* 0.160). Moreover, there were statistically significant reduction in fentanyl consumption on both POD1 (*P* = 0.018) and POD2 (*P* = 0.001). This indicated that the A + I block may have better analgesic efficacy compared to the S + F block. However, given the small population of analyzed patients after exclusion, the statistical power may be affected. In this study, we discovered that the analgesic efficacy of the A + I regimen was similar to or higher than that of the S + F regimen. In addition, our A + I group exhibited reduced motor blockade because of the motor-sparing nature of the A + I regimen.

In a randomized controlled trial, Hussein et al. compared the analgesic effects of the A + I and S + F regimens [29]. They observed no significant difference in pain scores during the first 24 h after surgery. These conflicting results may be attributable to variations in anesthetic concentration. Specifically, Hussein et al. used 0.25% bupivacaine, whereas we used 0.5% ropivacaine, which may have exerted relatively strong analgesic effects. In addition, because the A + I regimen only partially blocks stimulus input during surgery, the amount of intraoperative fentanyl used was higher in the A + I group than in the S + F group in our study. By contrast, in the study of Hussein et al., no significant between-group difference was observed in the total amount of intraoperative fentanyl used.

Early rehabilitation can improve clinical outcomes after TKA. Post-TKA rehabilitation can reduce swelling, accelerate recovery, improve pain control, and enhance patient satisfaction [30, 31]. Analgesia after TKA can accelerate rehabilitation by alleviating postoperative pain, which delays functional recovery, extends hospital stay, and increases overall opioid use [28, 32]. Early ambulation after surgery is a key component of rehabilitation. Evidence suggests that early ambulation shortens hospital stay, improves knee function, minimizes hospitalization costs, and prevents deep vein thrombosis (DVT) [33]. Although the risk of post-TKA DVT is relatively low among Asian individuals, it remains a major postoperative concern [34]. Motor function recovery and effective postoperative analgesia are critical factors influencing early ambulation following TKA surgeries. Nerve block regimens that impair motor function—for example, sciatic nerve block and femoral nerve block—can affect lower-extremity muscle strength and delay ambulation. In our study, the earliest ambulation time was significantly shorter in the A + I group than in the S + F group. This finding is likely attributable to the motor-sparing nature of ACB and iPACK and the superior analgesic efficacy observed in the A + I group, as evidenced by the lower average NRS scores in PACU and on POD1. The finding highlights the superior analgesic efficacy of the A + I regimen, especially on POD1 and POD2. In addition, the degree and frequency of motor blockade were lower in the A + I group than in the S + F group. The motor-sparing nature of the A + I regimen likely contributed to muscle strength preservation in the lower extremities and facilitated early rehabilitation and ambulation. These findings are consistent with those of studies indicating that motor blockade resulting from sciatic nerve block and femoral nerve block prevents early ambulation [9, 35]. To the best of our knowledge, our study is the first to report that the A + I regimen is more effective than the S + F regimen in alleviating postoperative pain and accelerating ambulation.

We found that the ROM was slightly better in the A + I group than in the S + F group. This difference may be attributable to the improved control of pain during movement and the reduced prevalence of motor blockade in the A + I group. Studies involving various nerve block regimens have reported conflicting findings regarding flexion ROM on POD1. For example, Wang et al. revealed that femoral nerve block was associated with improved postoperative ROM [6]. However, Paul et al. reported no significant differences in ROM among nerve block regimens such as epidural analgesia, continuous femoral nerve block, single-shot nerve block, and PCA [36]. To the best of our knowledge, our study is the first to compare ROM on POD1 achieved with the A + I and S + F regimens. The reduced NRS scores for pain at rest and pain during movement in the A + I group may have led to the improved ROM in this group. Our findings are consistent with those of Zheng et al., who reported that, compared with the S + F regimen, the A + I regimen improved patients’ quadriceps strength, modified Bromage motor blockade scores, and walking distances [37].

In our study, we observed no significant between-group difference in the length of hospital stay. McKee et al. reported no significant difference in hospital stay between patients receiving ACB plus posterior capsule local infiltration and those receiving periarticular local infiltration alone [38]. Similarly, Holbert et al. identified no significant difference in hospital stay between patients receiving ACB alone and those receiving spinal anesthesia [39]. However, Thobhani et al. reported that the A + I regimen resulted in a 35% shorter hospital stay than did femoral nerve catheter block [15]. The length of hospital stay is influenced by multiple factors, such as patients’ general condition, postoperative adverse events, patients’ desire, hospitalization costs, and health-care payment systems. In Taiwan, the National Health Insurance covers most of the costs associated with hospitalization and surgery. Therefore, patients in Taiwan are more likely to have longer hospital stays compared to those in other countries, which may introduce bias in the evaluation of hospital stay.

This study has several limitations. First, its retrospective design and relatively small sample size may limit the strength of our conclusions. Second, the efficacy of nerve block was not evaluated. However, all nerve block procedures were performed with ultrasound guidance by attending anesthesiologists trained in peripheral nerve blocks. Third, the use of other analgesics was not documented. However, the similarity in PCA readjustment rates between the two groups suggests similar rescue analgesic needs. Fourth, the assessment of motor function recovery was incomplete. We only analyzed the patients’ Bromage motor blockade scores on POD1 (approximately 12–24 h after surgery). We did not monitor the postoperative progression of motor blockade, which prevented us from comparing motor blockade after POD2 in the two groups. Fifth, potential inter-assessor variability may influence the outcome measure of motor function and ambulation time. Sixth, there was a significant difference in intraoperative analgesics use between groups. To better adjust the confounding factors, we performed subgroup analysis by excluding patients receiving fentanyl more than 150 µg and ketorolac. The results still revealed significant differences in NRS score and reduction of fentanyl consumption on POD1 and POD2. Significant between-group differences were still noticed regarding the motor function analysis in subgroup analysis. The results of significant pain reduction at POD2 in A + I group in all analysis may highlight the superior analgesic efficacy of A + I block. Finally, other functional factors, such as quadriceps muscle strength and walking distance, were not compared between the A + I and S + F groups.

## Conclusion

In conclusion, our findings indicates that the A + I regimen surpasses the S + F regimen in terms of analgesic efficacy, postoperative opioid requirement, ROM on POD1, degree of motor blockade and the time to earliest ambulation.

## Data Availability

Data sets generated during the current study are available from the corresponding author on reasonable request.

## Abbreviations

ACB: Adductor canal block
iPACK: Infiltration between the popliteal artery and the capsule of the knee
TKA: Total knee arthroplasty
POD: Postoperative day
PACU: Postanesthetic care unit
ROM: Range of motion
PCA: Patient-controlled analgesia
PSM: Propensity score matching
NRS: Numeric rating scale
DVT: Deep vein thrombosis
